# Poplar GTL1 Is a Ca^2+^/Calmodulin-Binding Transcription Factor that Functions in Plant Water Use Efficiency and Drought Tolerance

**DOI:** 10.1371/journal.pone.0032925

**Published:** 2012-03-02

**Authors:** Hua Weng, Chan Yul Yoo, Michael J. Gosney, Paul M. Hasegawa, Michael V. Mickelbart

**Affiliations:** Department of Horticulture and Landscape Architecture, Center for Plant Environmental Stress Physiology, Purdue University, West Lafayette, Indiana, United States of America; University of Massachusetts Amherst, United States of America

## Abstract

Diminishing global fresh water availability has focused research to elucidate mechanisms of water use in poplar, an economically important species. A GT-2 family trihelix transcription factor that is a determinant of water use efficiency (WUE), PtaGTL1 (GT-2 like 1), was identified in *Populus tremula × P. alba* (clone 717-IB4). Like other GT-2 family members, PtaGTL1 contains both N- and C-terminal trihelix DNA binding domains. *PtaGTL1* expression, driven by the *Arabidopsis thaliana AtGTL1* promoter, suppressed the higher WUE and drought tolerance phenotypes of an Arabidopsis *GTL1* loss-of-function mutation (*gtl1-4*). Genetic suppression of *gtl1-4* was associated with increased stomatal density due to repression of Arabidopsis *STOMATAL DENSITY AND DISTRIBUTION1* (*AtSDD1*), a negative regulator of stomatal development. Electrophoretic mobility shift assays (EMSA) indicated that a PtaGTL1 C-terminal DNA trihelix binding fragment (PtaGTL1-C) interacted with an *AtSDD1* promoter fragment containing the GT3 box (GGTAAA), and this GT3 box was necessary for binding. PtaGTL1-C also interacted with a *PtaSDD1* promoter fragment via the GT2 box (GGTAAT). *PtaSDD1* encodes a protein with 60% primary sequence identity with AtSDD1. *In vitro* molecular interaction assays were used to determine that Ca^2+^-loaded calmodulin (CaM) binds to PtaGTL1-C, which was predicted to have a CaM-interaction domain in the first helix of the C-terminal trihelix DNA binding domain. These results indicate that, in Arabidopsis and poplar, GTL1 and SDD1 are fundamental components of stomatal lineage. In addition, PtaGTL1 is a Ca^2+^-CaM binding protein, which infers a mechanism by which environmental stimuli can induce Ca^2+^ signatures that would modulate stomatal development and regulate plant water use.

## Introduction

*Populus* species have many commercial uses such as lumber, composite materials, paper pulp, and woody perennial landscape plants [1]. More recently, poplar has been identified as a potentially important source of plant biofuels [2]. Poplars, especially interspecific hybrids, are among the fastest biomass-producing plants in temperate latitudes [3]. *Populus tremula × P. alba* cuttings grown in fields can reach an average height of 4.5 m in a 3-year period [4]. However, the growth rate of poplar is highly dependent on soil water availability [5], [6], as even moderate water deficit causes significant reductions in biomass accumulation, substantially limiting the yield potential of hybrid poplars [7]. However, hybrid poplars exhibit genetic potential for enhanced water use efficiency (WUE) [7], which is the amount of biomass produced per unit of water used [8]. Recent advances in poplar molecular genetics make it feasible to access allelic variation for loci within the *Populus* genus that could enhance WUE [9]. However, little is known about specific genetic determinants that are responsible for WUE and drought tolerance in poplar.

More than 70% of plant transpiration occurs through stomatal pores formed by guard cells in the leaf epidermis [10], driven by the vapor pressure gradient between the sub-stomatal cavity and the ambient atmosphere [11]. Thus, the vast majority of plant transpiration is regulated either by controlling stomatal movement (opening and closing) or stomatal density [12]. Changes in stomatal aperture occur rapidly in response to phytohormones (e.g., ABA) and environmental factors such as light, photoperiod, CO_2_ concentration, humidity, and water deficit [11], [13], [14]. Stomatal opening and closing are processes that enable rapid control over transpiration in response to environmental changes such as those that occur during a diurnal day-night cycle [15], [16].

Stomatal development is a process that has been well characterized genetically [17]. The basic components of this developmental process are a cell lineage pathway involved in guard cell meristem differentiation and development, and stomatal formation [17] that is controlled by a negative regulatory MAP kinase pathway, which is responsible for stomatal patterning [18]. Interestingly, stomatal development appears to be regulated by many factors that control stomatal opening and closing [19]–[21]; however, mechanisms by which stimuli mediate phenotypic plasticity for stomatal density are largely unknown [22]. Recent research has linked genes that modulate the negative regulatory pathway to altered stomatal density, transpiration, and WUE [23], [24]. We established that the GT-2 like 1 (GTL1) trihelix transcription factor is a stomatal development regulatory determinant that controls stomatal density through trans-repression of *SDD1* expression [24]. *SDD1* encodes a subtilisin-like protease that is implicated to process propeptides into signaling ligands that are negative regulators of stomatal density [25]. GT-2 family transcription factors characteristically have N- and C-terminal trihelix DNA-binding domains that interact with a GT cis element [26], [27], and GTL1 interacts specifically with the GT3 box in the *AtSDD1* promoter [24]. Loss-of-function *gtl1* mutations enhance WUE and drought tolerance due to a reduced transpiration rate that is correlated with a lowered stomatal density compared to wild-type plants [24]. However, *gtl1* does not appear to affect biomass production or carbon assimilation, which may be attributable to more efficient carbon uptake or fixation, or a moderate reduction in stomatal density that reduces transpiration but not carbon assimilation [24], [28], [29].

Ca^2+^ signatures occur in response to external and internal stimuli such as UV light, abiotic stresses including water deficit, CO_2_ concentration, and ABA [30], and are implicated as secondary messengers in abiotic stress signaling [31]. These signatures presumably are decoded by Ca^2+^ sensor proteins such as CaM, CaM-like proteins (CML), calcineurin B-like proteins (CBL), and Ca^2+^-dependent protein kinases (CDPK) [32]. Both cytosol and nuclear Ca^2+^ concentrations increased transiently in Arabidopsis seedlings and tobacco BY-2 cells exposed to mannitol-induced hyper-osmolarity [33], [34]. In addition, genes encoding CaMs and CMLs, including CaM4, CML9, CML37, CML38, and CML39 were induced by water deficit [35], [36], [37]. Therefore, Ca^2+^-CaM signaling is inferred to play a role in acclimation or adaptation to water deficit stress. Plants contain numerous CaM-binding transcription factors such as TGA3, CAMTAs, MYB2, WRKY7, and CBNAC, and interaction between CaM and these transcription factors enhances or reduces their trans-activity, although the specific mechanisms involved are not known [38]–[40]. The transcription factors CAMTA1 (calmodulin-binding transcription activator 1) and CAMTA3 interact with Ca^2+^-activated CaM, activate gene expression, and positively mediate low temperature signaling and cold tolerance [41], [42].

Functional sufficiency results presented herein indicate that PtaGTL1 regulates WUE and drought tolerance based on ectopic expression in Arabidopsis. *PtaGTL1* expression repressed *AtSDD1* expression and the PtaGTL1 C-terminal trihelix DNA-binding domain (PtaGTL1-C) interacted directly with an *AtSDD1* promoter fragment through the GT3 box. In addition, PtaGTL1-C interacted with the GT2 box in a *PtaSDD1* promoter fragment. These findings establish PtaGTL1 as an ortholog of AtGTL1 and indicate that the regulatory pathway controlling stomatal development is conserved in Arabidopsis and poplar. Moreover, PtaGTL1-C was determined to bind CaM in a Ca^2+^-dependent manner. Therefore, PtaGTL1 could potentially be involved in the decoding of a Ca^2+^ signature induced by environmental stimuli, such as water deficit, into the regulation of stomatal density and plant water use.

## Results and Discussion

### Identification and structure of a poplar GT-2 family member, PtaGTL1

**Figure 1:**
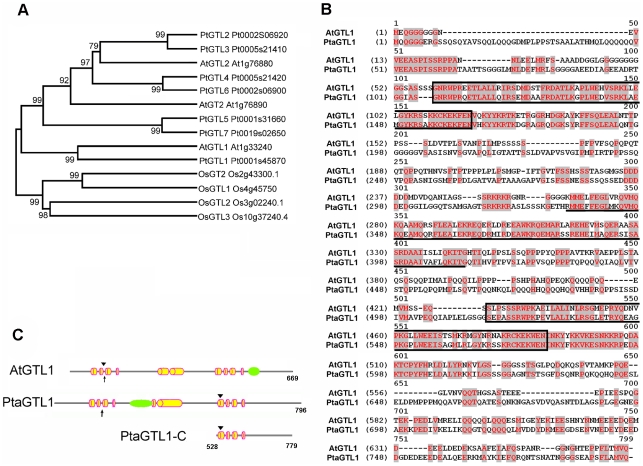
*In silico* sequence analyses identify PtaGTL1 as an AtGTL1 ortholog. A. Phylogenetic analysis of GT-2 transcription factors of *Arabidopsis thaliana*, *Oryza sativa*, and *Populus trichocarpa* was performed by the neighbor joining method using MEGA 5.0 with 1000 boot strap replicates [43]. B. Protein sequences of AtGTL1 and PtaGTL1 were aligned with the CLUSTW program. Fragments corresponding to the N- and C-terminal DNA binding domains are enclosed in boxes and the conserved central helical region is underlined. C. Schematic diagrams of AtGTL1 and PtaGTL1. Yellow cylinders represent predicted helices (http://www.compbio.dundee.ac.uk/www-jpred/index.html); arrowheads identify the position of predicted CaM-binding sites (http://calcium.uhnres.utoronto.ca/ctdb/ctdb/home.html); arrows identify the position of nuclear localization sequences (http://cubic.bioc.columbia.edu/cgi/var/nair/resonline.pl); green ellipses identify the position of PEST sequences (http://emboss.bioinformatics.nl/cgi-bin/emboss/epestfind). The PtaGTL1-C fragment (from amino acid residue 528 to 779 of PtaGTL1) that was used for promoter and CaM binding assays is also illustrated.

A BLASTp search analysis was conducted on proteins encoded in the *Populus trichocarpa* genome (http://www.phytozome.net) and seven proteins were identified based on primary sequence similarity and domain topology with AtGTL1 ([24]; Figure 1). Topological comparisons focused on the N- and C-terminal trihelix DNA-binding domains and an intervening central helix region of about 70 amino acids. These seven *P. trichocarpa* proteins were designated as PtGTL1 to PtGTL7 with PtGTL1 sharing the highest identity with AtGTL1 and decreasing similarity with AtGTL1 in PtGTL2 through 7 ([43]; Figure 1A). Interestingly, PtGTL1 is more similar to AtGTL1 than the closest paralog in Arabidopsis, AtGTL2 (Figure 1A), which has overlapping functions with AtGTL1 (unpublished data). In addition, all rice GTL (OsGTL) homologs were phylogenetically different from all AtGTL and PtGTL proteins (Figure 1A). The phylogenetic analysis indicates that PtGTL1 is a potential ortholog of AtGTL1.

Primers were designed from the *PtGTL1* sequence for PCR-amplification of *GTL1* using genomic DNA isolated from leaves of the *Populus tremula × P. alba* clone 717-IB4, a poplar hybrid with high transformation efficiency [44]. This gene was designated as *PtaGTL1*. A 3196 bp *PtaGTL1* genomic DNA fragment from the translation start codon to the codon immediately upstream of the stop codon was amplified and the sequence was deposited into the NCBI GenBank (accession number BankIt1459934 Seq1 Jn113092). The genomic fragment contained two introns (a 635 nt intron located 426 nt downstream of the translational start codon and a 135 nt intron located 282 nt upstream of the stop codon) that may facilitate intron-mediated enhancement of gene expression [45], [46]. PtaGTL1 is 95% identical to PtGTL1 (not shown) and has 80 and 82% identity with AtGTL1 in the amino (N)- and carboxy (C)-terminal trihelix DNA-binding domains, respectively (Figure 1B). GT-2 transcription factors from Arabidopsis and rice all have a central conserved region between the N- and C-terminal trihelix domains [47]. PtaGTL1 also has such a region that shares 74% identity with that of AtGTL1. Although the function of this conserved central region is unknown, in rice it is implicated to affect GT-2 interaction with GT boxes [26]. In addition, PtaGTL1 has other putative domains that exist in AtGTL1 (Figure 1C). A nuclear localization signal (NLS) is located between the second and third helices of the N-terminal trihelix domain. Both PtaGTL1 and AtGTL1 contain a CaM-binding site and a PEST sequence, although these two domains are located in different regions of the two proteins. The CaM-binding site is located in the C-terminal trihelix domain of PtaGTL1, whereas it is located in the N-terminal trihelix domain of AtGTL1 (http://calcium.uhnres.utoronto.ca/ctdb/ctdb/home.html). Thus, it is predicted that the C-terminal DNA-binding domain of PtaGTL1 may have a similar function to the N-terminal DNA-binding domain of AtGTL1 [48]. The PEST sequence is proposed to be a target for proteolysis [49]. The carboxy-terminal location of the AtGTL1 PEST sequence suggests that it may serve as a constitutive signal for protein degradation, whereas the PtaGTL1 PEST sequence location between the two trihelix domains may be indicative that it is a conditional signal for proteolysis [50], [51].

### *PtaGTL1* expression causes extragenic suppression of the Arabidopsis *gtl1-4* trichome phenotype

To investigate the biological function of PtaGTL1, the 3196 nt *PtaGTL1* genomic DNA fragment was inserted into the binary vector pCAMBIA1302 in-frame with an *mGFP* gene [52]. The *35S* promoter of pCAMBIA1302 was replaced with the 2.9 kb *AtGTL1* promoter [24], producing *AtGTL1pro:PtaGTL1-GFP*. This plasmid was used to transform *gtl1-4* plants by the floral dipping method [53]. The AtGTL1 promoter fragment was sufficient for suppression of *gtl1-4* [24].

The first generation of transgenic plants (T1) was subjected to selection for hygromycin resistance. Plants of eleven independent hygromycin-resistant T1 lines were selected for trichome branch length similar in size to wild type, in contrast to the larger trichome branch length of *gtl1-4* plants [54]. Homozygous T3 plants of lines 8, 9, and 19 were used for further analyses. Transgenic lines harboring the pCAMBIA1302 vector without *AtGTL1pro:PtaGTL1-GFP* (hereafter, referred to as vector control plants) had similar trichome branch lengths as *gtl1-4* plants (data not shown).

**Figure 2:**
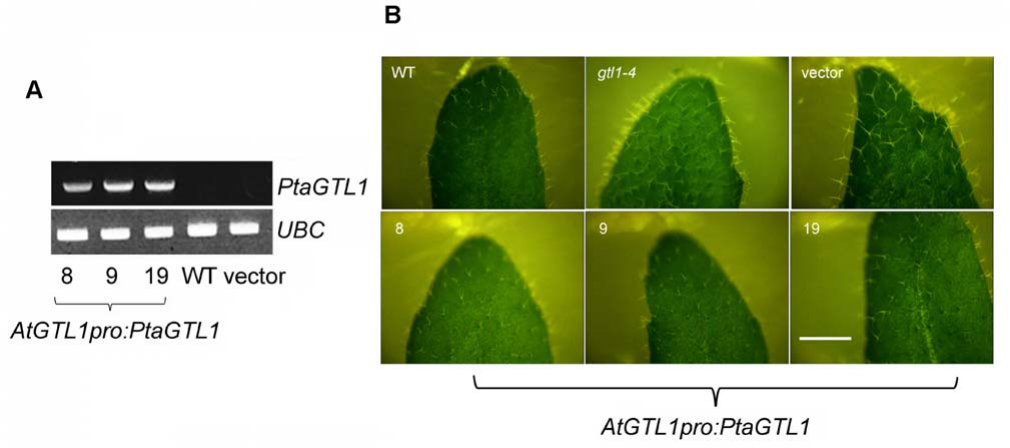
*AtGTL1pro:PtaGTL1* expression suppresses the leaf trichome phenotype of *gtl1-4*. WT is Col-0; vector is a transgenic line expressing *pCAMBIA1302* in *gtl1-4*; 8, 9, and 19 are three transgenic lines expressing *AtGTL1pro:PtaGTL1* in *gtl1-4*. A. *PtaGTL1* transcript abundance in 4-week-old plants was detected by RT-PCR with *PtaGTL1* gene-specific primers (PtaGTL1 F2 and PtaGTL1 R2). UBC (ubiquitin conjugating enzyme 21, At5g25760) was used as an internal standard. B. Bright-field images, taken under a dissecting microscope, illustrate trichomes on the adaxial surface of fully expanded rosette leaves of 4-week-old plants. Bar indicates 4 mm.

*PtaGTL1* transcript was detected with gene specific primers in plants of lines 8, 9, and 19, but was undetectable in wild-type and vector control plants (Figure 2A). *AtGTL1pro:PtaGTL1-GFP* expression, similar to *AtGTL1pro:AtGTL1-GFP* expression [24], suppressed the larger trichome branch length caused by *gtl1-4* (Figure 2B), an indication that the poplar gene can genetically suppress the mutation.

### *PtaGTL1* regulates plant water use by modulating stomatal density

**Figure 3:**
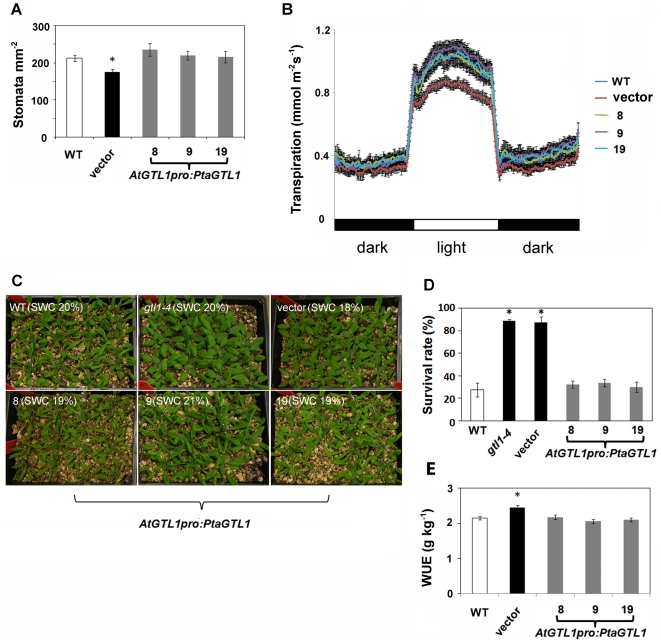
*AtGTL1pro:PtaGTL1* expression suppresses the stomatal density, drought tolerance, and WUE phenotypes of *gtl1-4*. A. Stomatal density (number of stomata per mm^2^) of leaf abaxial epidermis (fully expanded rosette leaves) from 45-day-old plants (12/12 hr photoperiod) was determined (mean ± SE, n = 3). Asterisk indicates that the mean value of vector plants is significantly different from that of wild type at P<0.05 (Student's t-test). B. Diurnal transpiration rates of 5-week-old plants grown in a growth chamber (12/12 hr photoperiod) were determined gravimetrically (mean ± SE, n = 4). C. Plant water-deficit tolerance of 3-week-old plants (16/8 hr photoperiod) was evaluated in four 15 cm×20 cm containers per genotype (20 plants per container). Representative containers for each genotype were photographed 12 days after withholding water and relative soil water content (SWC) is indicated in brackets. SWC equals water content 12 days after withholding water/water content at saturation. D. Survival rate was determined after 14 days without watering and 4 days after re-watering (mean ± SE, n = 4 containers, 20 plants per container). After 14 days without water, all genotypes had the same relative SWC, WT, 15.3±2.2 g; *gtl1-4*, 15.3±2.0 g; vector, 14.0±1.8 g; #8, 15.4±2.8 g; #9, 16.4±1.2 g, and 14.3±1.5, mean ± SEM. E. Integrated WUE_B_ under water-sufficient conditions was determined over a period of 3 weeks, starting with 17-day-old plants. Water loss throughout the growth period was determined by weighing containers before and after each irrigation. Total water loss is the sum of three determinations (WT, 62.9±2.8 g; vector, 54.3±3.0 g; #8, 60.7±4.0 g; #9, 61.6±2.7 g; and #19, 64.2±3.3 g, mean ± SEM). Shoot dry weight was determined at the completion of the experiment (WT, 126±7 mg; vector control, 122±6 mg; #8, 124±10 mg; #9, 117±5 mg; and #19, 125±6 mg, mean ± SEM). WUE_B_ was calculated as shoot dry weight divided by total water loss (mean ± SE, n = 12 to 15). Asterisk indicates that WUE_B_ of vector plants is significantly different from that of wild-type plants at P<0.05 (Student's t-test). All 3 lines of *gtl1-4* expressing PtaGTL1 have the same WUE_B_ as wild-type plants.

The suppression of the *gtl1-4* trichome phenotype of *gtl1-4* led us to investigate whether PtaGTL1 may also regulate stomatal density, transpiration, drought tolerance, and WUE. Forty-five-day-old *gtl1-4* vector control plants grown under a 12 hr photoperiod have a 17% reduction in abaxial stomatal density compared with wild-type (213±9 stomata per mm^2^), while there was no statistical difference in abaxial stomatal density between *gtl1-4* expressing *PtaGTL1* and wild-type plants (Figure 3A). Thus, *PtaGTL1* expression suppressed the abaxial stomatal density phenotype in *gtl1-4*. Reduced daytime transpiration associated with the *gtl1-4* was also abrogated by *PtaGTL1* expression (Figure 3B). Diurnal transpiration was assessed over a 36 hr period by gravimetric analysis. Plants were grown under water-sufficient conditions under a 12 hr photoperiod. *gtl1-4* plants expressing *PtaGTL1* exhibited ca. 15% greater peak light period transpiration rates than *gtl1-4* vector control plants (Figure 3B), and water loss was 21% higher over a 24 hr period (data not shown). Together, these results indicate that PtaGTL1 regulates stomatal density, which affects transpiration.

Drought tolerance of 3-week-old plants was evaluated by withholding water commencing immediately after the soil was uniformly irrigated to saturation. After 12 days and at a relative soil water content of ca. 20%, transgenic plants expressing *PtaGTL1* and wild type plants exhibited severe leaf wilting symptoms while leaves of *gtl1-4* plants were fully turgid (Figure 3C). Fourteen days post irrigation (relative soil water content of 14.0±1.8% to 16.4±1.2%, not statistically different), when leaves of all plants were no longer turgid, plants were irrigated and survival was determined 4 days after re-watering. *PtaGTL1* expression decreased survival of *gtl1-4* plants from 89% to 30–34% (Figure 3D). Collectively, these results indicate that PtaGTL regulation of stomatal density affects transpiration and drought tolerance.

Integrated WUE (WUE_B_) of *gtl1-4* vector control plants (2.4±0.06 g kg^−1^) was approximately 14% higher than wild type (2.1±0.05 g kg^−1^) (Figure 3E) in water sufficient conditions. By contrast, all three *PtaGTL1* expressing transgenic lines had WUE_B_ similar to wild type (Figure 3E). Dry weights of vector control, wild-type, and *PtaGTL1* plants were not statistically different (WT, 126±7 mg; vector control, 122±6 mg; #8, 124±10 mg; #9, 117±5 mg; and #19, 125±6 mg, mean ± SEM, respectively). However, total water loss of *gtl1* vector control plants was significantly lower than that of wild type, while all three PtaGTL1 transgenic lines exhibited similar water loss to wild type (WT, 62.9±2.8 g; vector, 54.3±3.0 g; #8, 60.7±4.0 g; #9, 61.6±2.7 g; and #19, 64.2±3.3 g, mean ± SEM, respectively). These results indicate that PtaGTL1 is a determinant of WUE_B_.

### Nuclear-localized PtaGTL1 trans-represses *SDD1* expression

**Figure 4:**
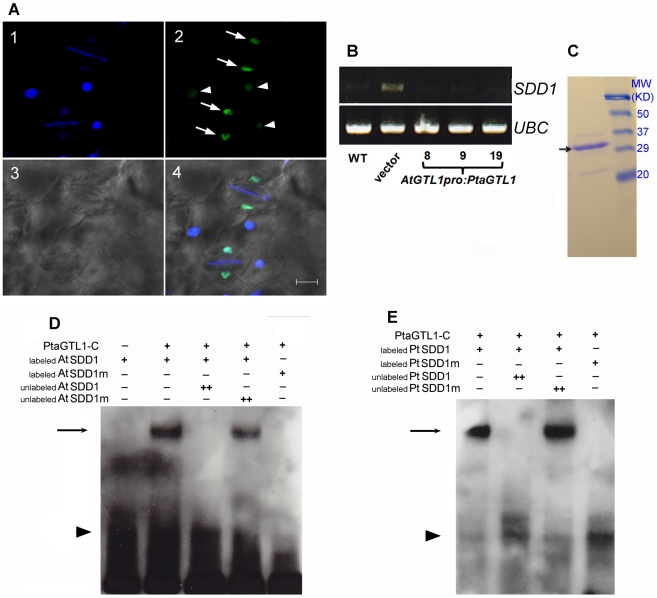
PtaGTL1 is localized to the nucleus, binds to a GT3 box-containing *AtSDD1* promoter fragment, and trans-represses *AtSDD1* expression. A. An abaxial epidermal layer of a one-week-old seedling leaf from a transgenic *gtl1-4* plant expressing *AtGTL1pro:PtaGTL1-GFP* (line 9) was photographed with a confocal laser scanning microscope. Nuclei of guard cells (arrows) and pavement cells (arrowheads) were stained with DAPI (4′,6-diamidino-2-phenylindole). 1, DAPI fluorescence, showing nuclear location; 2, GFP fluorescence, showing PtaGTL1 localization; 3, Light microscopic picture of the corresponding cells; 4, Panels 1, 2, and 3 merged. Bar equals 10 µm. B. *SDD1* expression in fully expanded rosette leaves from 5-week-old plants (12-h diurnal photoperiod) was determined by RT-PCR. UBC was used as an internal control. C. Two micrograms of affinity purified 6×His-PtaGTL1-C was separated by SDS-PAGE (15% Tris-HCl gel). The arrow indicates the 6×His-PtaGTL1-C band (31 kDa), molecular markers are on the right. D. and E. PtaGTL1-C interaction with *AtSDD1* and *PtSDD1* promoter fragments require the GT3 and GT2 boxes, respectively. Recombinant 6×His-PtaGTL1-C was used in an EMSA with biotin-labeled DNA probes (200 ng, +) corresponding to a fragment in the *AtSDD1* promoter harboring the GT3 box (GGTAAA) (D) or a fragment in the *PtSDD1* promoter harboring the GT2 box (GGTAAT) (E). Mutant versions of the SDD1 promoters (SDD1m), in which CC was substituted for GG in the GT3 (*AtSDD1*) and GT2 (*PtSDD1*) boxes, were used to test the necessity of the GT3 and GT2 boxes in the interaction. Unlabeled probes of both original and mutated DNA fragments (1000 ng, ++) were used as competitors to test the binding specificity. Arrows and arrow heads indicate the positions of protein-promoter probe complexes and free probes, respectively.

*PtaGTL1* expression reduced *AtSDD1* transcript abundance in fully expanded leaves of *gtl1-4* plants compared to that of wild type (Figure 4B), indicating that PtaGTL1 is a negative regulator of *SDD1* expression. PtaGTL1-GFP expression was detected in a subcellular compartment of both guard and pavement cells in the abaxial epidermis (Figure 4A) and co-localized with DAPI staining (Figure 4A, panels 1 and 4). These results indicate that PtaGTL1 is a *SDD1* trans-repressor (Figure 4A).

The C-terminal DNA-binding domain fragment of PtaGTL1 (PtaGTL1-C, 252 amino acids, Figure 1B) was purified (Figure 4C) for use in an electrophoretic mobility shift assay (EMSA) with a 28 nt *AtSDD1* promoter fragment that includes the GT3 box (GGTAAA) [24]. PtaGTL1-C caused a mobility shift of the biotin labeled At*SDD1* promoter fragment, which did not occur when the unlabeled *SDD1* fragment was added as a competitor (Figure 4D). In contrast, an unlabeled *SDD1* promoter fragment with mutations to the first two nucleotides of the GT3 box (GGTAAA →CCTAAA) did not reduce the gel shift caused by the interaction between PtaGTL1-C and the labeled *SDD1* fragment (Figure 4D). Furthermore, the mutated *SDD1* promoter fragment labeled with biotin did not interact with PtaGTL1-C (Figure 4D). These results confirm that Pta-GTL1-C trans-represses Arabidopsis *SDD1*.

*Populus trichocarpa* has a putative SDD1 (PtSDD1) protein that is 60% identical to AtSDD1. In the *PtSDD1* promoter region (3000 nt upstream of the *PtSDD1* start codon), no GT3 or GT1 box was identified. Instead, there is a GT2 box (GGTAAT) residing 934 nt upstream of the start codon. An EMSA determined that PtaGTL1-C physically interacted with a 23 nt promoter fragment of *PtSDD1*, which contains the GT2 box (Figure 4E). A GGTAAT→CCTAAT mutation to the promoter fragment inhibited this interaction (Figure 4E). These results confirm that PtaGTL1-C binds to the poplar *SDD1* promoter through an interaction that requires the GT2 box.

PtaGTL1 is a poplar ortholog of AtGTL1, and a regulator of stomatal density, transpiration, drought tolerance, and WUE. PtaGTL1 interacts with the *PtSDD1* promoter through the GT2 box. Together with gain-of-function results in Arabidopsis, it appears that PtaGTL1 is a trans-repressor of *SDD1* expression in poplar, although we cannot exclude the possibility that PtaGTL1 in poplar may play a different role from that of AtGTL1. Recently, a poplar (*Populus nigra ×* (*P. deltoides × P. nigra*)) ortholog of *ERECTA* was identified [23]. ERECTA is a leucine-rich repeat receptor-like kinase that functions downstream of SDD1 to regulate stomatal development [55]. Overexpression of poplar *ERECTA* suppressed the higher stomatal density phenotype caused by *er-105*, an Arabidopsis *ERECTA* loss-of-function mutation. Together, these results indicate the conservation of key determinants in the negative regulatory pathway of stomatal lineage in Arabidopsis and poplar.

There is genetic potential to enhance poplar WUE by reducing stomatal density [56], [57], although mechanistic determinants have not been identified. *PtaGTL1* and poplar *SDD1* may be such deterministic loci. This knowledge base may provide insight for molecular breeding or biotechnological approaches to improve poplar drought tolerance and water use efficiency while maintaining yield. Since poplar is often cultivated under irrigated conditions, it is important that genotypes exhibit higher WUE at soil water field capacity and low to moderate water deficit to minimize transpirational water loss. Both *PtaGTL1* and *PtaSDD1* are loci for which allelic variation may lead to higher WUE with no or negligible reduction in biomass production.

### PtaGTL1 C-terminal DNA-binding domain interacts with CaM in a Ca^2+^-dependent manner

**Figure 5:**
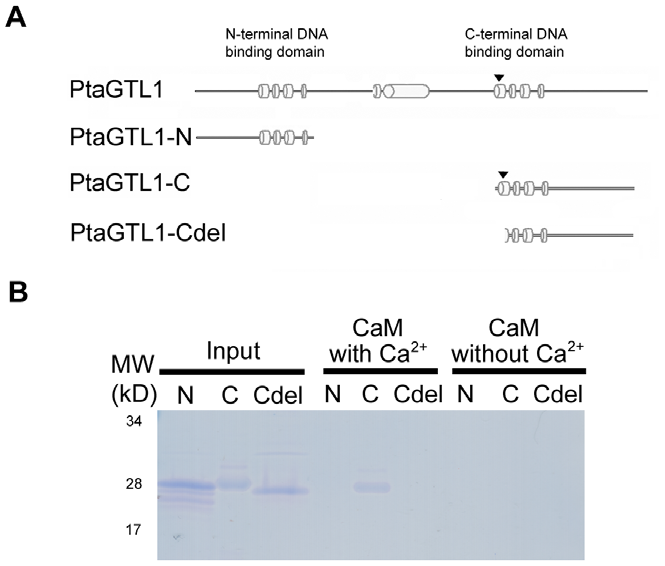
PtaGTL1-C interacts with CaM in a Ca^2+^-dependent manner. A. Schematic diagrams of PtaGTL1 fragment proteins: PtaGTL1-N, PtaGTL1-C, and PtaGTL1-Cdel fragments are illustrated. PtaGTL1-N and PtaGTL1-Cdel fragments do not include CaM-binding site. Arrowheads indicate the position of putative CaM-binding site. B. Ten micrograms of purified PtaGTL1-N, PtaGTL1-C, and PtaGTL1-Cdel were incubated with CaM-agarose in a buffer solution with or without 2 mM CaCl_2_. After extensive washing with the buffer solution, the proteins bound to CaM-agarose were eluted with elution buffer containing 10 mM EGTA and separated by SDS-PAGE (12% Tris-HCl separating gel). Inputs indicate 10 µg of protein fragments used for CaM-binding assays.

PtaGTL1 is predicted to have a CaM-binding site that is located in the first helix of the C-terminal DNA-binding domain (Figure 5A; http://calcium.uhnres.utoronto.ca/ctdb/). To determine if PtaGTL1 interacts with CaM *in vitro*, purified PtaGTL1-C (Figure 5B) was incubated with CaM-agarose from bovine testes with or without Ca^2+^. Mammalian CaM has been successfully used for identifying plant CaM-binding proteins [58]–[60]. A protein band was detected in a fraction eluted from CaM-agarose incubated with PtaGTL1-C and 2 mM CaCl_2_ (Figure 5B; lane 5). By contrast, no proteins were obtained in eluate from CaM-agarose incubated with PtaGTL1-C without Ca^2+^ (Figure 5B; lane 8). Furthermore, fragments that do not contain the predicted CaM-binding site (PtaGTL1-N and PtaGTL1-Cdel) did not bind to the CaM-agarose in either condition. Together, these results indicate that PtaGTL1 interacts with first helix of the C-terminal DNA-binding domain, and the interaction is Ca^2+^-dependent.

**Figure 6:**
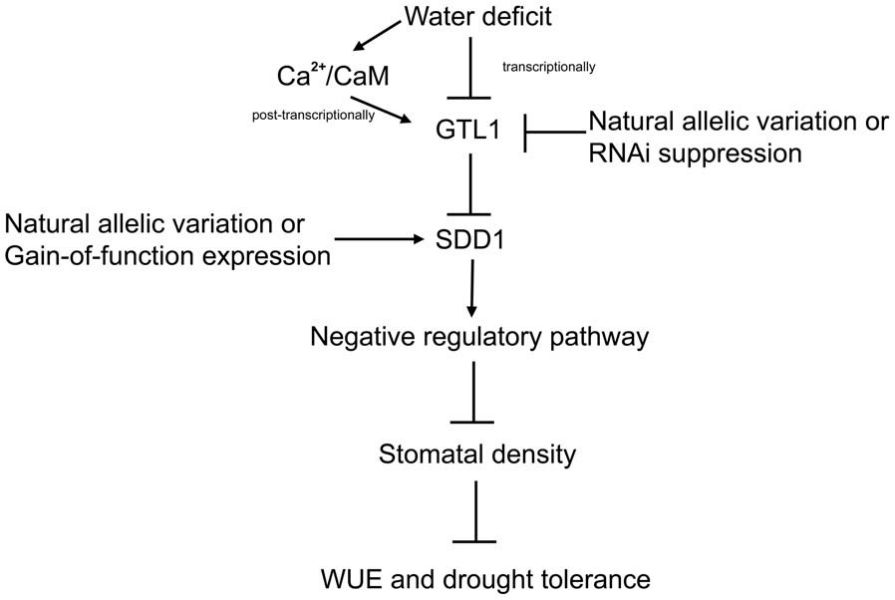
A model for GTL1 as a node for integration of the environment on stomatal development, WUE, and drought tolerance. GTL1 activity is negatively regulated by water deficit conditions in two ways. Transcriptionally, GTL1 is down-regulated under water deficit conditions. Post-translationally, GTL1 activity is repressed through Ca^2+^/CaM signaling induced by water deficit. Lower GTL1 activity will lead to increased stomatal density through reduced trans-repression of SDD1, a negative regulator of stomatal development. Therefore, GTL1 serves as a node to integrate environment signals into regulation of stomatal density, thus achieving higher WUE and drought tolerance. Allelic variation in poplar *GTL1* and *SDD1* may be used to identify poplar with higher WUE and no or negligible reductions in biomass. Alternatively, RNAi suppression or other biotechnological approaches can be used to generate commercial genotypes that have higher WUE without a reduction in biomass and are potentially more drought tolerant.

Ca^2+^ loaded CaM binding to PtaGTL1-C infers that PtaGTL1 is involved in the decoding of a stimulus-induced Ca^2+^ signal. PtaGTL1 negatively regulates *SDD1* expression; consequently, it may be that CaM binding to PtaGTL1 modulates the activity of the transcription factor to trans-repress *PtaSDD1* expression. It is known that hyperosmotic stress and dehydration cause cytosolic and nuclear Ca^2+^ transients [33], [34]. We posit that water deficit induces a Ca^2+^ signature that is decoded by CaM through physical interaction with PtaGTL1, resulting in higher *SDD1* expression that reduces stomatal density and transpiration and enhances drought tolerance and WUE (Figure 6). These results suggest that water deficit signaling regulates PtaGTL1 activity by both transcriptional and posttranscriptional mechanisms, and identifies the transcription factor as a node for integration of water deficit signaling into a stomatal development pathway. Future work will provide greater physiological insight about the significance of PtaGTL1 as a regulator of plant water use in adaptation and crop production.

## Materials and Methods

### Plant materials and growth conditions

The primary *Arabidopsis thaliana* genotypes were wild type (Columbia (Col-0) ecotype) and *gtl1-4* (SALK_005972). Seeds were stratified for 2 days at 4°C in the dark and then sown onto soilless media (2∶1 mixture of ProMix PGX soilless media (Premier Horticulture) and Turface calcined clay (Profile Products)). Plants were maintained under a 16/8 hr photoperiod and 60% relative humidity unless otherwise noted, and were illuminated with fluorescent and incandescent lights (photosynthetic photon flux density: 120 µmol m^−2^ sec^−1^). The temperature was maintained at 22°C [light]/20°C [dark].

### Generation of transgenic plants

A 3196 bp DNA fragment corresponding to the whole genomic sequence encoding *PtaGTL1* (including introns) was amplified from DNA of *Populus tremula × P. alba* (clone 717-IB4) leaves, using the following primers: PtaGTL1 F1: 5′- CCATGGATGCAACAAGGAGGTGG-3′; PtaGTL1 R1: 5′- GCGGTAATCTAACCATGGCCAAAAAGGAGG-3′. It was then cloned into a modified vector pCAMBIA 1302, generating an in-frame fusion with the N-terminus of mGFP. The 35S promoter in pCAMBIA 1302 was replaced with the *AtGTL1* promoter [24]. The resultant plasmid was mobilized into *Agrobacterium tumefaciens* (GV3101 strain), which was used to transform *gtl1-4* by the floral dip method [53].

The first generation of transgenic plants (T1) was selected for hygromycin resistance. Eleven hygromycin-resistant T1 plants with trichome branches similar in length to wild type were selected for further analyses. Progeny of four independent T1 lines (8, 9, 13, and 19) that exhibited a hygromycin resistance ratio of 3∶1 were identified (#8: 42 of 167 hygromycin sensitive, χ^2^ = 0.002, P>0.95; #9: 40 of 172 hygromycin sensitive, χ^2^ = 0.28, P>0.55; #13: 45 of 190 hygromycin sensitive, χ^2^ = 0.18, P>0.65; and #19: 56 of 211 hygromycin sensitive, χ^2^ = 0.27, P>0.60). These four lines were assumed to have a single functional T-DNA insertion that results in hygromycin resistance and *PtaGTL1* expression. T3 progeny of these lines were evaluated to identify genotypes that were homozygous for the T-DNA insertion.

### RNA extraction and reverse transcription (RT)-PCR analysis

Total RNA from fully expanded rosette leaves was extracted using TRIzol (Invitrogen), according to the manufacturer's instructions. PCR was performed using cDNA synthesized from 1 µg of total RNA by the ThermoScript RT-PCR system (Invitrogen) using oligo-dT primer. The same amount of cDNA was used for PCR analysis using the following primers: for *PtaGTL1* (F2: 5′- AGTGGCAATAGGTGGCCAAGGCAAGAA -3′; R2: 5′- GCATCCCTGCTGAGATTTCTTCCCAAAG -3′), for *AtSDD1* (F: 5′- GAAAGCGATAAAGGATGG -3′; R: 5′- GGTTACAGAGATTGGACTTC -3′), for *UBC* (F: 5′- ATACAAAGAGGTACAGCGAG -3′; R: 5′- TTCTTAGGCATAGCGGCG -3′). Amplification was performed using ExTaq™ (Takara), cycle numbers: 27 (*UBC*), 35 (*PtaGTL1*), or 40 (*SDD1*).

### Physiological analyses

All physiological analyses including water deficit survival, gravimetic water loss, and integrated WUE were carried out using methods described in Yoo et al. (2010) with slight modifications [24]. Analysis of water deficit stress tolerance was conducted on 3-week-old plants (20 per container, 4 containers per genotype) grown in soilless media in 15 cm×20 cm (1 L) containers. Containers were irrigated to saturation and weighed at the beginning of the experiment (initial soil fresh weight). After 14 days, containers were weighed (final soil fresh weight). Then plants were re-watered and the survival rate was evaluated 4 day after re-watering. Plants that survived the treatment were green with actively growing tissues. The dry weight was determined after the pots with media were completely dried in an oven. Relative soil water content (SWC) was calculated as (final soil fresh weight – soil dry weight)/(initial soil fresh weight – soil dry weight)×100%.

Transpiration rate was determined gravimetrically for plants grown under water sufficient conditions. Plants were grown in soilless media (200 mL containers) under a 12/12 hr photoperiod for 5 weeks. Prior to water loss measurements, containers were covered with a polyethylene wrap to prevent evaporation from the surface of the media. Each container was placed onto a balance and weight was recorded to a laptop computer every 5 minutes for 36 hours. Leaves were excised and photographed, and leaf area was determined using the ImageJ program (National Institutes of Health). Transpiration rate was expressed as mmol H_2_O m^−2^ s^−1^.

Integrated WUE_B_ was calculated as shoot dry weight divided by total water loss over a period of 4 weeks. Plants were grown individually in a plastic container covered with a lid that had a 0.5 cm hole in the center into which the seed was sown. This container design completely sealed the root zone of the plant so that water loss was only possible from the rosette. Plants were grown under a 12/12 hr photoperiod and watered once per week starting from 3 days after sowing (DAS). Starting at 17 DAS, each container was weighed before and after each irrigation to determine water loss. The last measurement was taken 38 DAS. Rosettes were excised and dried at 37°C to a constant weight to determine shoot dry weight. WUE_B_ was calculated by dividing shoot dry weight by total water loss (mean ± SE, n = 12 to 15).

### Stomatal density determination

A leaf surface imprint method was used to determine stomatal density [61]. Abaxial epidemal cell outlines of rosette leaves of 45-day-old plants (12/12 hr photoperiod) were imprinted onto cyanoacrylate droplets that were placed onto glass slides. For each genotype, three fully expanded rosette leaves from three independent plants were used for imprinting. Images were taken under 200× magnification using a Nikon-OptiPhot2 microscope. Stomata were counted in an area of 0.069 mm^2^. For each leaf, four independent areas were counted and the average of these four was considered as one replicate.

### EMSA

EMSA analysis was used to determine the *in vitro* interaction between PtaGTL1-C protein and DNA fragments in the *AtSDD1* and *PtSDD1* promoters. Single-stranded complementary oligonucleotides corresponding to a region of the *AtSDD1* promoter that included a GT3 box and a region of the *PtSDD1* promoter that included a GT2 box were synthesized (MWG Operon). For the *AtSDD1* promoter, the two complementary oligonucleotides were: 5′-TTCTTTGGCTTGGTAAAACTTCAATGGA -3′ and 3′-AAGAAACCGAACCATTTTGAAGTTACCT-5′. The two complementary oligonucleotides of the *PtSDD1* promoter were: 5′-TTCGTGAATATGGTAATGATTAT-3′ and 3′-TTCGTGAATATGGTAATGATTAT-5′. The underlined sequences correspond to the GT3 and GT2 boxes, respectively. Oligonucleotides were labeled using the Biotin 3′-end DNA labeling kit (Thermo Fisher Scientific). To generate double-stranded probes, biotinylated complementary oligonucleotides were boiled for 5 min in TE buffer (10 mM Tris-HCl and 1 mM EDTA, pH 8.0), then cooled slowly to room temperature overnight. Unlabeled complementary oligonucleotide pairs were also annealed to make double-stranded competitor probes. The EMSA reaction was carried out using a Light Shift Chemiluminescent EMSA kit (Thermo Fisher Scientific), according to the method described previously [24].

### Protein purification and CaM binding assay

For recombinant protein purification, a cDNA fragment coding for PtaGTL1-N, PtaGTL1-C, and PtaGTL1-Cdel (Figure 5) were amplified and cloned into pENTR TOPO (Invitrogen). The primers used for PCR amplification were: PtaGTL1-N For, 5′-CACCCAACAAGGAGGTGGAGAAAG-3′ and PtaGTL1-N Rev, 5′-CTAGCTGATTCCAACCGGGGCA-3′; PtaGTL1-C For, 5′-CACCAGCGAACCAGCATCATCAAG-3′ and PtaGTL1-C Rev, 5′-CTACTTGTACGCCATTTTCCTCTCC-3′; PtaGTL1-Cdel For, 5′- CACCCAAGAAGCCGGGCCTAAG-3′ and PtaGTL1-Cdel Rev, 5′-CTACTTGTACGCCATTTTCCTCTCC-3′. cDNA fragments were subcloned by Gateway LR recombination into pDEST17 (Invitrogen), generating an in-frame fusion with a hexahistidine (6×His) tag at the N-terminus. The resulting plasmid was chemically transformed into BL21 *E. coli.* strain for protein expression and purification (Invitrogen).

The 6×His-PtaGTL1-C fusion protein was expressed in BL21 *Escherichia coli* cells after induction with 0.4 mM of isopropyl β-D-thiogalactopyranoside for 4 h at 37°C. The culture was harvested by centrifugation for 15 min at 4000 *g*. Total protein extracts were obtained using the CelLytic™ B plus kit, according to the manufacturer's instructions (Sigma catalog no. CB0050). His-tagged proteins were then purified using the HIS-Select® HF Nickel Affinity Gel system according to the manufacturer's instructions (Sigma catalog no. H0537).

A CaM binding assay was performed according to the method described in [62], [63]. The 6×His-PtaGTL1-C peptide (10 µg) was incubated with 100 µl CaM−agarose (Sigma catalog no. P4385) in 10 mM Tris-HCl buffer (pH7.5) without or with 2 mM CaCl_2_ for 2 h at room temperature. CaM−agarose was then washed with 500 µl of binding buffer (without or with CaCl_2_, correspondingly) five times. Then, CaM-binding proteins were eluted with elution buffer (10 mM Tris-Cl, 10 mM EGTA) and used for SDS-PAGE analysis.
